# Report of Departmental Committee on Sickness Benefit Claims

**Published:** 1915-02-06

**Authors:** 


### NATIONAL INSURANCE ACT DEVELOPMENTS.

### Report of Departmental Committee on Sickness Benefit Claims.

In an article which appeared in The Hospital for January 9, page 337, under the above heading we dealt with the first part of the Report, which had particular reference to the sickness claims in men's societies. The remedy suggested is that the societies should be encouraged to exercise greater care in the supervision of claims more particularly by the development, and in some instances the institution, of sick-visiting. With regard to the action of the doctors the remedy suggested has already taken shape in the form of a uniform system of certification. It is hoped that these measures may result in a substantial reduction in the excessive claims on men's societies.

### Women's Insurance.

It was well known that the most serious excess of claims for sickness benefit was in respect of women's societies, but it is somewhat startling to learn from the Report that "except in certain societies, in which, for example, domestic servants or women of the semi-professional class have been aggregated, the amount expended in respect of women considerably exceeds the actuarial provision." The Committee assign several causes for this excess. In the first place it has to be pointed out that in the absence of any reliable information as to the sickness of women the actuaries based their original estimates on the assumption that the rates of sickness would be the same as those applicable to men.

Ignorance of the principles of insurance was one of the reasons given for the excess of claims in men's societies, but this is an even more potent cause in the case of women. Again, among the insured women there is a very large proportion of ill-paid and ill-fed persons, and this must of necessity add to the sickness experienced. Moreover, 7s. 6d. a week bears a larger proportion to the average woman's wage than 10s. does to the average man's wage, so that although double insurance is not so much in evidence among women as it is among men, there is the same inducement to prolong the claims for benefit. In this connection also the Committee point out that a larger proportion of the male than of the female insured population have families dependent upon them. Another special cause is the difficulty of supervising the behaviour of women who are in receipt of benefit, the possibility of doing ordinary housework or, at appropriate seasons, extraordinary housework, being an inducement to women to stay on the funds longer than they otherwise would. Above all these causes, however, there is the one predominating cause—namely, that of illnesses accompanying pregnancy. The difficulty of dealing with illnesses of this character as the ground of claims for sickness benefit has caused endless trouble. The Committee now point out that the only question is whether the woman is rendered incapable of work, and the fact that the incapacity is, in popular language, "due to pregnancy" is irrelevant. Special attention is directed to this aspect of the question in the recommendations made by the Committee.

They also add "that the main cause of the excess lies in the fact that the incidence of sickness among women employed in industrial occupations generally is heavier than was anticipated, or could have been calculated, when the actuarial estimate was framed."

### Suggested Remedies: Women's Insurance.

The suggested remedies for dealing with the excessive claims disclosed by the evidence undoubtedly form the most interesting section of the Report, more particularly as regards the far-reaching proposals in connection with women's insurance.

To combat the evil of the prevailing ignorance among women of the principles of insurance, it is suggested that women should take a more active share in the work of managing and administering the societies in which they are insured. As a concrete proposal, it is submitted that women should be included in the membership of all committees concerned with the administration of women's benefits.

With regard to sickness due to pregnancy, it has already been indicated that the Committee held that the only question to be considered in regard to a claim for benefit is whether the woman is thereby incapacitated for work. It is admitted that the decision of this question is frequently a matter of peculiar difficulty, and as a means of lessening that difficulty it is suggested "that in the last month of pregnancy, when it may be assumed, as a general rule, without further inquiry, that the woman should not go to her work, it should be assumed that she is in fact incapacitated, and that an automatic payment should be made to her in respect of the last month upon the statement, supported by a medical certificate, that she is at that stage of pregnancy." The Committee point out that this is an extension of the benefits granted to women under the Act which the present contributions will not bear. They therefore suggest that such payments as are made under this heading should be treated separately, and that the necessary funds should be obtained from a special Parliamentary grant. It is insisted that the societies should not be allowed to make any profit from receipts from this special fund, and the principle that each society should retain an interest in the administration of its general benefit funds, in order that, if possible, on an actuarial valuation a surplus may be disclosed, is emphasised as being of the highest importance to the general welfare of the societies.

The proposal to institute a special "pregnancy sickness fund" would not, however, be sufficient to obviate the disclosure of a deficiency on the women's societies as a whole, and the question of reducing the rate of benefit or increasing the rate of contribution had to be considered. Such alteration the Committee describe as obviously impossible, but they make the alternative suggestion that solvency may be restored by a redistribution of the proportions in which the weekly contributions are at present divided as between the amounts paid to the societies and the amounts applied towards the reduction of the initial reserve fund. In other words, it is proposed to reduce the amount of the sinking fund and thereby to prolong the period which must elapse before the additional benefits indicated in the Act can be contemplated. Thus the young people of both sexes of the present and future generations of insured persons would be penalised more than under the existing system in a strictly equitable distribution of the funds, for the benefit of the older insured persons who came within the scope of compulsory insurance at the inception of the Acts. These suggestions are undoubtedly the most important recommendations made by the Committee, and it will be interesting to note whether the Government will act upon them by the introduction of amending legislation in the present session. In view of the disclosures of excess claims for benefit contained in the evidence of witnesses it is clear that some action must be taken without delay to prevent the disclosure of deficiencies in the majority of women's societies when the first valuations come to be made in July next.

### The Medical Service.

The Committee make some further suggestions, which, though of less importance, are nevertheless interesting. Thus, for instance, they indicate that members of approved societies have been slow in putting into operation the machinery provided under the Act for the decision of disputes with their societies, and it is suggested that this is, at least in part, due to the ignorance of the members of the rules of the societies to which they belong. It is thus proposed that it should be incumbent upon each society to furnish a copy of its rules, free of charge, to all insured persons when they join the society. Similar remarks are made as to the utilisation of the machinery of complaint by the approved societies against medical practitioners, and the attitude of the societies in preferring general statements of condemnation against the doctors instead of substantiating specific cases is strongly deprecated.

The adequacy of the medical service also calls for comment, and while the Committee are satisfied that in general the doctors have had little difficulty in obtaining for their patients, where necessary, treatment outside the scope of medical benefit, they state that there is evidence that in certain quarters the hospital accommodation is inadequate, and that sickness benefit claims are thereby increased. It is further pointed out that the institution of a system of nursing in serious cases would be an advantage in securing the quicker return to health of insured persons, and would thus benefit the funds of the societies, but, apart from possible special Parliamentary grants, there is no money available for the purpose.

### Miss MacArthur's Memorandum.

So far the findings and recommendations of the Committee are unanimous, but while agreeing that financial readjustment is imperative, Miss Mary R. MacArthur is of opinion that the proposals made are inadequate, and she gives expression to her own recommendations in a memorandum appended to the Report which cannot be ignored. She believes that the suggestions made by the Committee would only have a temporary effect in relieving the situation, and that a fundamental alteration of the scheme is necessary to make it National Health Insurance in fact as well as in name. "The evidence shows," she says, "that serious difficulties, hardships, and anomalies are inevitable so long as the Act is administered by approved societies, as at present constituted."

With regard to women's insurance in particular she expresses the opinion that the provisions of the Act require considerable amendment before they will meet the special needs and requirements of women. In this connection she urges that the whole question of the care, treatment, and provision before, during, and after confinement should be the subject of an immediate inquiry by a Royal Commission on Maternity which should be appointed with comprehensive terms of reference, and that the Report should be made with the least possible delay. Certain specific reforms are suggested, and the arguments used are developed with striking lucidity. Her main conclusion is that the principal anomaly of the Act is the present arrangement for the valuation of the societies and declaration of surplus and deficits. It is unfair, in her opinion, that a compulsorily insured person who is in no way responsible for the deficit which his society may show on valuation should, by some accident of choice in joining a particular society approved by the Government, be subjected to a reduction of benefit. The result of this opinion is that she recommends the organisation by the Insurance Commissioners of a National Society with (*a*) branches coincident with existing areas of the local insurance authorities, (*b*) rules framed by the Commissioners, and (*c*) benefits administered by the Insurance Committees under the control of the Commissioners. It is not proposed immediately to disorganise the existing approved societies—vested interests would make this impossible—but the National Society would in course of time predominate, as it would be composed of (*a*) any insured person who is not a member of an approved society—thus in effect solving the question of the Deposit Contributor class, (*b*) any insured person hereafter dropping out of an approved society for any reason whatever and not within one month being accepted by another, (*c*) any person desiring to transfer to such society, and (*d*) members of societies from which approval has been withdrawn.

This is a far-reaching proposal and it is certain to command attention. Whether it is possible for the Government to adopt it remains to be seen.

